# A Three-Dimensional Computational Model of Collagen Network Mechanics

**DOI:** 10.1371/journal.pone.0111896

**Published:** 2014-11-11

**Authors:** Byoungkoo Lee, Xin Zhou, Kristin Riching, Kevin W. Eliceiri, Patricia J. Keely, Scott A. Guelcher, Alissa M. Weaver, Yi Jiang

**Affiliations:** 1 Department of Mathematics and Statistics, Georgia State University, Atlanta, Georgia, United States of America; 2 School of Physics, Graduate University of Chinese Academy of Science, Beijing, China; 3 Laboratory for Optical and Computational Instrumentation, Laboratory for Cell and Molecular Biology and Department of Biomedical Engineering, University of Wisconsin-Madison, Madison, Wisconsin, United States of America; 4 Department of Cell and Regenerative Biology, University of Wisconsin-Madison, Madison, Wisconsin, United States of America; 5 Department of Chemical and Biomolecular Engineering, Vanderbilt University, Nashville, Tennessee, United States of America; 6 Department of Cancer Biology, Vanderbilt University Medical Center, Nashville, Tennessee, United States of America; University of California, Berkeley, United States of America

## Abstract

Extracellular matrix (ECM) strongly influences cellular behaviors, including cell proliferation, adhesion, and particularly migration. In cancer, the rigidity of the stromal collagen environment is thought to control tumor aggressiveness, and collagen alignment has been linked to tumor cell invasion. While the mechanical properties of collagen at both the single fiber scale and the bulk gel scale are quite well studied, how the fiber network responds to local stress or deformation, both structurally and mechanically, is poorly understood. This intermediate scale knowledge is important to understanding cell-ECM interactions and is the focus of this study. We have developed a three-dimensional elastic collagen fiber network model (bead-and-spring model) and studied fiber network behaviors for various biophysical conditions: collagen density, crosslinker strength, crosslinker density, and fiber orientation (random *vs.* prealigned). We found the best-fit crosslinker parameter values using shear simulation tests in a small strain region. Using this calibrated collagen model, we simulated both shear and tensile tests in a large linear strain region for different network geometry conditions. The results suggest that network geometry is a key determinant of the mechanical properties of the fiber network. We further demonstrated how the fiber network structure and mechanics evolves with a local formation, mimicking the effect of pulling by a pseudopod during cell migration. Our computational fiber network model is a step toward a full biomechanical model of cellular behaviors in various ECM conditions.

## Introduction

Extracellular matrix (ECM), the extracellular part of multicellular structure, not only provides mechanical support and physical separation to tissues [1], [2], but also regulates key biological processes including development, differentiation, and wound healing [3]–[5]. ECM dynamically communicates with cells by chemical and mechanical signals [6]–[10]. Moreover, as a major component of the tumor microenvironment, the ECM regulates cancer cell proliferation and invasion into the stroma [11], [12]. In breast cancer, tumor tissue is found to be stiffer than normal tissue. Collagen, the main component of ECM in the breast, is observed be denser in breast tumor tissue [12]–[14]. The role of stromal collagen deposition in cancer is a topic of recent intense study, due to the association with aggressive cancer behaviors [11]–[16].

Tumor initiation and progression has been linked to perturbations in stromal collagen [11]. Recent evidence from both human and animal studies indicate that increased density and alignment of breast tissue, derived from deposition and/or crosslinking of collagen, may paradoxically increase the formation and aggressiveness of breast cancer [12], [15]. Specifically, the collagen fibers surrounding tumors are believed to be mechanically stretched, locally deformed, and realigned perpendicular to the tumor boundary [16]. The resulting collagen structures, named tumor associated collagen signatures (or TACS), can be used as independent biomarkers that predict breast cancer progression [12], [16]. Both *in vitro* and *in vivo* studies suggest that radially aligned collagen fibers facilitate cancer cell invasion out along the realigned fibers [16]. Despite these observations, we do not understand the mechanisms of the causality and interactive relationship between the tumor associated collagen and tumor cell migration.

A collagen gel consists of collagen fibers, interconnected into a three-dimensional fiber network. The basic structural unit of collagen is a triple-helix, tropocollagen, of 300 nm in length and 1.5 nm in width. Multiple tropocollagen molecules form collagen fibrils, via covalent cross-linking. Multiple collagen fibrils form collagen fibers, which cross-link to form a 3D network of collagen matrix. The mechanical properties of single collagen fiber is well understood [17]–[20]. Molecular weight and size of a collagen fibril [17], length and thickness of a collagen fiber [18], as well as the tensile modulus [19] and bending modulus [20] of a fiber have been measured experimentally. The bulk mechanical properties of collagen gels have also been reported extensively [21]–[23]. How collagen structure relates to its mechanical properties, on the other hand, has enjoyed less attention [16], [23]. Only recently has the tensile modulus of an aligned collagen network been determined relative to a randomly organized collagen network [24]. Moreover, the inter-fiber crosslinker that binds fibers into a network structure is poorly understood. Experimentally, lysyl oxidase can be added to collagen to build covalent crosslinks [12], but this type of crosslinker is generally thought to be between fibrils to hold together larger intra-fiber structure. Collagen can be cross-linked using a chemical reagent, e.g. glutaraldehyde [21], [23], but we do not know if this fixative agent can recapitulate the natural collagen crosslinkers. All of these contribute to our lack of understanding of collagen (and other ECM) at the intermediate scale between single fiber and bulk gel. This intermediate scale is precisely the cell scale. Hence, understanding of collagen at this scale is important to understanding cell-ECM interactions.

Theoretical and computational models have been developed to study ECM mechanics from a single molecule to fiber network, and tissue level. Buehler et al. used atomistic molecular dynamics (MD) to determine the mechanical properties of a single collagen molecule [25] and a collagen fibril [26], [27]. However, the mechanical property of a collagen gel is different from that of individual collagen fibrils, showing nonlinear elastic behavior and strain-stiffening [28]. Rubinstein and Panyukov described the nonlinear elasticity by a nonaffine deformation of network chain model [29]. Stein et al. used a worm-like-chain network to reproduce the strain stiffening of a fiber network [30]. Head et al. showed nonaffine and bending dominated regime and affine and stretching dominated regime in semiflexible polymer networks using a 2D model [31]. Zahalak et al. built a tissue model, composed of cells and ECM, and predicted mechanical properties of cell and ECM using relaxation tests [32]. Chandran and Barocas used a micromesh fiber network model in 2D and showed the nonlinear mechanical stress-strain responses for the affine model and network model [33]. Onck et al. and Huisman et al. pointed out that the fiber realignment and network architecture directly influences nonlinear elasticity in semiflexible polymer networks, such as F-actin networks, in 2D [34] and 3D models [35]. Because our goal is to understand how cells interact with collagen networks with various collagen densities and different connectivity and geometry conditions, neither atomic molecular dynamics, nor continuous models will work.

To build a computational model of collagen networks that help us to understand the properties collagen at the cell scale, we have developed a 3D off-lattice, elastic fiber network model. This model is similar to that of Stein et al. [30], who extracted the connectivity and geometry feature of a collagen fiber network from actual microscopy images, and modeled crosslinker as a torsional spring between fibers with one single parameter. In order to easily alter the fiber network connectivity and geometry conditions, we generate random fiber networks with each crosslinker as explicit elastic springs connecting fibers. Conceivably, the crosslinkers are a combination of covalent and non-covalent interactions. Covalent chemical bonds between fibers are strong, short-ranged, and non-breakable under the type of external forcing we consider. Non-covalent interactions, including van der Waals interactions and viscous drag between fibers, are longer-ranged but diminish at long distance, a.k.a. the bonds would break when the fibers are further apart. Hence our elastic treatment is a reasonable first order approximation for the combined effect of both covalent and non-covalent bonds. Assuming that the crosslinker strength and density do not change as collagen changes density, we can fit for crosslinker strength and density using the same experimental shear data from Stein et al. [30]. We then simulate various fiber network connectivity structures with different parameter conditions. The model allows us to investigate how local deformation propagates through the fiber network.

## Results

### Computational model of collagen network based on experimental fiber-scale parameters and gel-scale structure

**Figure 1:**
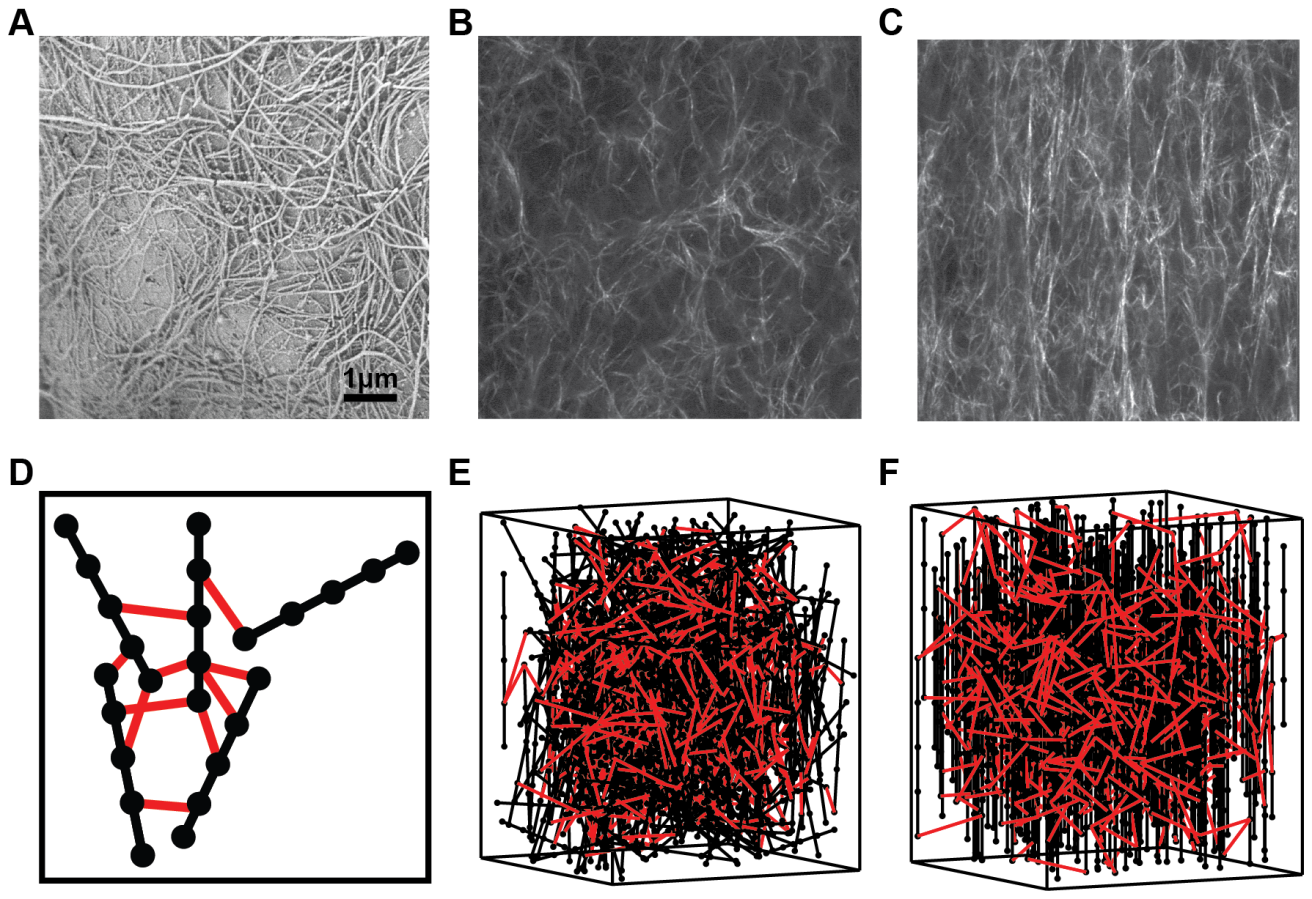
Collagen fibers. (A) SEM image of collagen fibers from a normal mouse mammary gland. (B) Representative SHG images of *in vitro* 2 mg/ml collagen gel under 3% strain (B) and 30% strain (C). (D) Schematic illustration of a bead-and-spring collagen fiber network model. Black lines represent collagen fibers, and red lines represent crosslinkers. Black dots represent beads, which have elastic connection with other beads. Constructed fiber network model with 1 mg/ml density and 2× total number of fibers [N] crosslinker density, random network (E) and prealigned network (F).

Figure 1A shows a scanning electron microscopy image of the intertwined collagen fibers forming a network *in vivo*. From such images, we measured the length and width distribution of the collagen fibers using ImageJ (data not shown), which informed us the choices of the fiber dimensions. We used second harmonic imaging techniques to visualize the fiber network structure. The initially random fiber orientation of the 2 mg/ml collagen gel (figure 1B) becomes aligned in the direction of the external force (figure 1C), after 30% strain. Figure 1D shows a schematic illustration of our elastic bead-and-spring fiber network model. Black lines represent collagen fibers and red lines represent crosslinkers. Black dots are beads, which can have elastic interaction with other beads. The bead-bead distance, or the length of springs, should correspond to the persistence length of the collagen fiber. Increasing the number of beads per fiber can simulate more realistic spatial configuration but exponentially increases simulation time. Because the main characteristic of individual collagen fibers is elasticity [25]–[27], we modeled individual collagen fibers as elastic springs. Between beads on different fibers, we added elastic springs to model inter-fiber crosslinking interactions. We also allowed multiple crosslinkers to connect the same bead, as shown in figure 1D. Therefore, the crosslinkers in our model have two adjustable parameters: the crosslinker density and the crosslinker strength. The crosslinker density is in the unit of total number of collagen fibers (N). When we add the same number of crosslinkers as the total number of collagen fibers, the crosslinker density is 1N. This way we can build fiber networks from sparsely crosslinked to densely crosslinked by varying the density parameter. The crosslinker strength parameter corresponds to the crosslinker stiffness. In addition, to examine how fiber geometry alters the network mechanical properties, we examined two different geometrical structures of collagen fibers: randomly oriented fibers (figure 1E) and prealigned fibers uniformly in the vertical direction (figure 1F).

**Figure 2:**
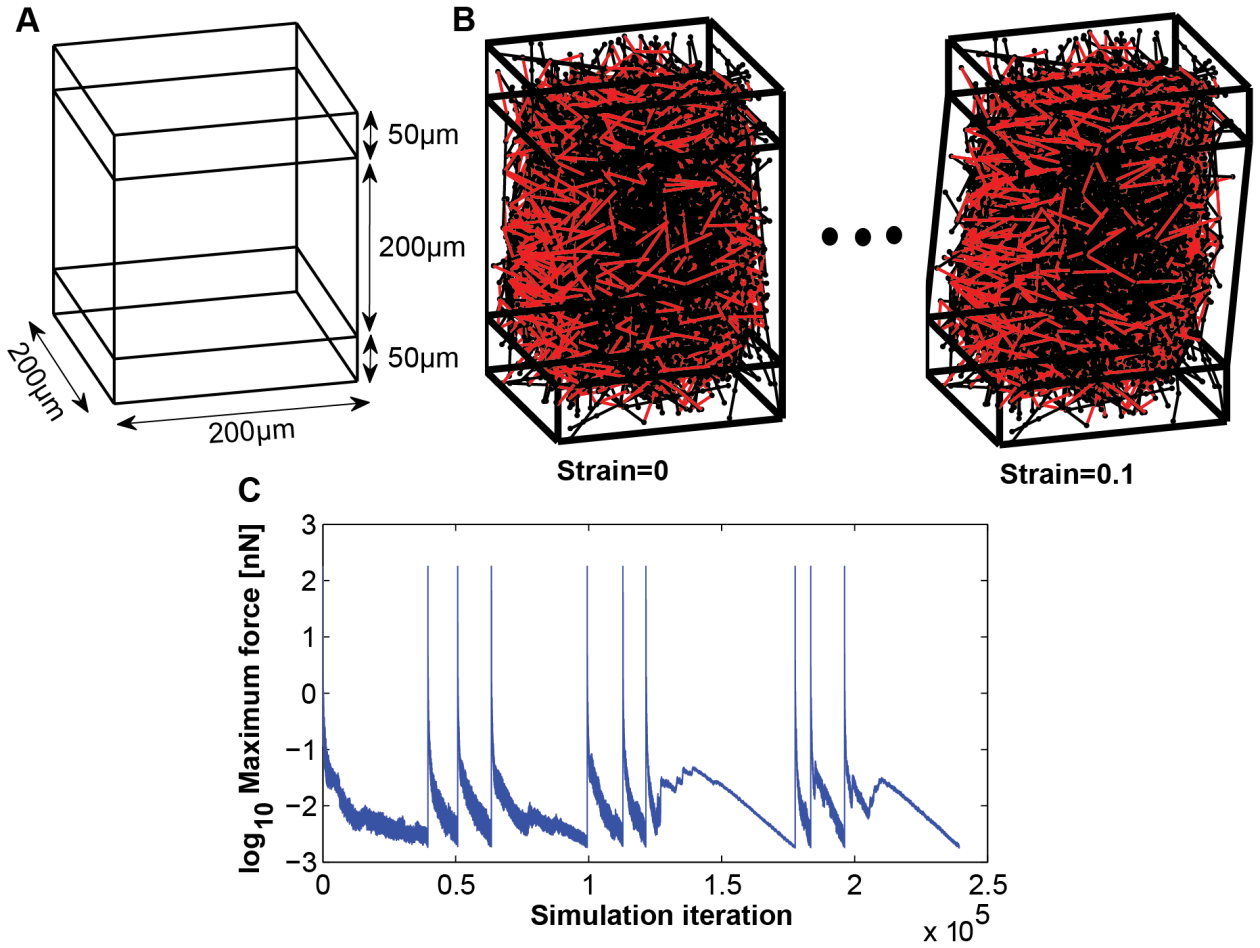
Shear simulation test using elastic fiber network model. (A) The simulation box is 200 µm (length) ×200 µm (width) ×300 µm (height). The bottom 50 µm and top 50 µm of the box are anchored region. The beads in the bottom anchored region are fixed and the beads in the top anchored region are deformed to y-axis. (B) Snapshot images for initial and quasi-equilibrium state of 0.1 shear strain with 2 mg/ml collagen density, 8N crosslinker density, 400 KPa crosslinker strength, and random fiber network. The shear strain step size is 0.01 (2 µm) and total ten shear strains are applied to the simulation box. (C) Maximum force of ten shear strain test from 0.01 strain to 0.1 strain, assuming that the fiber network reaches the quasi-equilibrium state when the maximum force decreases the below of 10^−5^ of the maximum value at each deformed state.

For simplicity, we specify that collagen fibers have homogeneous length and thickness. Collagen type I fibers are the most abundant collagen in ECM, typically 20 µm–200 µm in length and 200 nm–350 nm in thickness [18]. We first fixed collagen size parameters, 100 µm in length and 0.3 µm in diameter, based on the quantitative analysis of SEM images of collagen in mouse mammary glands (figure 1A). Given that the molecular weight of a single collagen fibril is 8.05×10^5^, and the typical single fibril is 300 nm in length and 1.5 nm in diameter [17], we calculated the total number of fibers in the simulation box for different collagen densities (1–4 mg/ml). The bead number per a fiber is 5, for feasible computing cost. We set the maximum available crosslinker-binding number per bead to 10 and the initial available crosslinker-binding distance is from 0.45 µm to 50 µm. After generating the initial fiber configuration in a simulation box, we connect crosslinkers randomly between two beads on different fibers. We vary four different collagen densities (1, 2, 3, 4 mg/ml), two different geometries (random *vs.* prealigned), 16 different crosslinker strengths (50–800 KPa, with a 50 KPa increment), and eight different crosslinker densities (2–16N, with 2N increments) for shear tests, with 5 independent runs for each parameter set. The simulation box is 200 µm (length) ×200 µm (width) ×300 µm (height). Fibers within the top and bottom 50 µm in the simulation box are anchored, as illustrated in figure 2A, which is based on our simulation tests for various anchored depths in figure S1. In the shear tests, all beads in the bottom anchor region are fixed in space, while all beads in the top anchor region are fixed in relative positions and are moved as a ‘solid’ without deformation for each strain step, in the direction of y-axis. For the tensile test, all beads in the top and bottom anchor regions are fixed as ‘solids’ and move in the opposite directions along z-axis by half of the strain step size. The network stress computation considers the center, unfixed simulation box only. Fibers have no interaction with the simulation box. Figure 2B shows the initial and the quasi-equilibrium states of a 2 mg/ml random fiber network at 0.1 shear strain.

The fiber network is described by its total elastic potential energy:
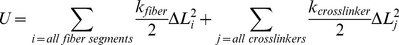
(1)


The elastic interaction between beads follows Hooke's law and the spring constant (*k*) is calculated by the Young's modulus of a collagen fiber (*E*), the fiber cross-sectional area (*A*), and the fiber segment length (*L*): *k = EA/L*. ΔL is the deformed length of either fiber or crosslinker. The Young's modulus of a collagen fibril in wet condition has been measured to be between 30 and 800 MPa [19], [36]. We use the fiber Young's modulus of 32 MPa based on the atomic force microscopy experiments [19]. The crosslinker in our model is the main parameter to adjust the whole fiber network connectivity and stiffness. Similar to the collagen fiber, the crosslinker is represented as purely elastic, and its strength is adjusted by altering the Young's modulus, while we set the cross-sectional area of a crosslinker to the same as that of the collagen fiber. Fixed and varying model parameters are shown in Table 1.

**Table 1:** Parameters for elastic collagen fiber network simulations.

Fixed Fiber Parameters		Varied Parameters	
Fiber length	100 µm [18]	Collagen density (mg/ml)	1, 2, 3, 4
Fiber diameter	0.3 µm [18]	Crosslinker density (x N)	2, 4, …, 16 (increment 2)
Fiber Young's modulus	32 MPa [19]	Crosslinker strength (KPa)	50, 100, …, 800 (increment 50 KPa)
Bead per fiber	5	Network structure	Random *vs*. Prealigned

We use the conjugate gradient method to search for the next fiber network structure with a lowest total potential energy. This method is an efficient alternative to Langevin dynamic simulations, which calculate the forces on each bead and integrate the equation of motion for each bead with small time steps, using either explicit [37] or implicit [38] integration methods. Langevin dynamics, while providing the realistic dynamics, is computationally expensive because the typical integration time step is very small from 1 µs to 1 ns, depending on the Young's modulus of a collagen fiber, the minimum fiber segment length, crosslinker strength, and the minimum crosslinker length. The conjugate gradient method, on the other hand, calculates the conjugate vector on each bead to quickly find the minimum energy state; it is an approach commonly used in molecular dynamics simulations to estimate a three dimensional folded protein structure [39]. We assume that the fiber network reaches the quasi-equilibrium state when the maximum force of fiber-bead system is reduced by five orders of magnitude of that in the strained state. In addition, we perform 5 replica simulations, each from a different initial fiber configuration, for each run, to ensure that our simulation of the fiber network is not trapped in a local energy minimum far from the global minimum.

### Identification of model parameters using shear tests in the small strain region

Collagen gel is a viscoelastic material, which has both elastic (strain-rate independent) and viscous (strain-rate dependent) features. This viscoelasticity is confirmed by *in vitro* collagen tensile stretching tests, which show that tensile modulus is strongly dependent on the strain rate [40]–[42]. Most *in vitro* experiments have used rather fast strain rates, 0.1–10 mm/min, compared with strain rates that are likely to be generated by mechanical interaction with migrating cells. Based on *in vivo* cell migration velocities of 0.01–0.1 µm/min [43], and lamellipodial extension rates of 1–10 µm/min [44], cell-collagen interactions should lead to a strain rate that is 3-5 orders of magnitude slower than those used in the tensile tests. For such slow strain rates, we can safely ignore the viscous aspects of collagen fiber networks. Thus, a purely elastic network model should be a reasonable approximation for our purpose. Because purely elastic moduli can be extracted from shear data, we used data from shear tests to parameterize our elastic model. We then used the parameterized model to predict tensile test results at zero strain rate and validated the result by comparing with experimental data at various strain rates. Our predicted Young's moduli for various collagen densities at a zero strain rate are very closed to those of fitted values at slow strain rates based on experimental data, validating our model.

Stein et al. [30] showed that collagen gels are softer in small strain regions (<0.1), but becomes increasingly stiffer as the strain region is larger than 0.1 using shear experiments for six different collagen densities (0.5, 1, 2, 3, 4, 5 mg/ml). The elastic modulus (G′) is constant for the small strain region and keeps increasing for larger strain [30]. Assuming that the viscous effect of collagen gels is negligible in the slow strain rate region that is relevant to cell migration, we focus on the elastic effect of collagen gels using our elastic fiber network model. We simulated shear tests for a small strain region from 0.01 strain to 0.1 strain as 0.01 strain step increment (Figure 2). Figure 2C shows the maximum force value during the shear simulation in figure 2B.

**Figure 3:**
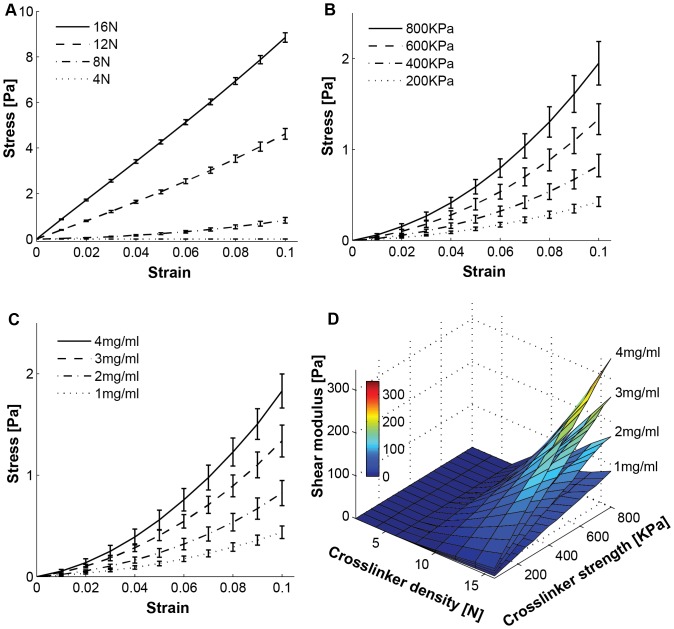
Shear simulation results. We simulated 8 different crosslinker densities (2, 4, …, 16N, 2N increment), 16 different crosslinker strengths (50, 100, …, 800 KPa, 50 KPa increment), and 4 different collagen densities (1, 2, 3, 4 mg/ml) for random fiber networks, which is total 512 different parameter sets. Shear stress - shear strain curves for ten strains using a 0.01 strain step size are shown in various crosslinker densities with fixed 400 KPa crosslinker strength (A) of 2 mg/ml collagen density case, various crosslinker strengths with fixed 8N crosslinker density (B) of 2 mg/ml collagen density case, and various collagen densities with fixed 400 KPa crosslinker strength and fixed 8N crosslinker density (C). Five independent runs were conducted for each parameter set. Only four curves for each varied parameter are shown for the better visualization. (D) Shear modulus surface plot for four different collagen densities, 8 different crosslinker densities and 16 different crosslinker strength. Each modulus value was calculated from the regression line slope of the stress-strain curve.

In order to find the crosslinker parameters using experimental shear test data, we performed shear simulations on random fibers networks of 512 parameter combinations (4 collagen densities, 16 crosslinker strengths, and 8 different crosslinker densities) as previously described. We did 5 independent simulations for each parameter combination by generating different random initial fiber networks. To show the effect of crosslinker density on gel properties, we plotted the stress-strain curves of four different crosslinker densities (4, 8, 12, 16N) for fixed 400 KPa crosslinker strength and 2 mg/ml collagen in figure 3A. To show the effect of crosslinker strength, we plotted the stress-strain curves of four different crosslinker strengths (200, 400, 600, 800 KPa) for fixed 8N crosslinker density and 2 mg/ml collagen in figure 3B. To determine how collagen fiber density affects stiffness properties of the network, we plotted the stress-strain curves of four different collagen densities (1, 2, 3, 4 mg/ml) for fixed 8N crosslinker density and 400 KPa crosslinker strength in figure 3C. From these stress-strain curves, it is clear that denser and stiffer crosslinkers increase the mechanical stiffness of the whole fiber network. Figure 3D shows that the shear modulus of the fiber network increases linearly as a function of the crosslinker strength, but increases nonlinearly as a function of the crosslinker density. We see a transition from a liquid like gel (shear modulus ∼0 Pa) to a linear elastic material at a crosslinker density around 8N. Furthermore, in regions with dense crosslinkers (>8N), the shear modulus depends linearly on the collagen density, as similarly occurs in experiments [30].

**Figure 4:**
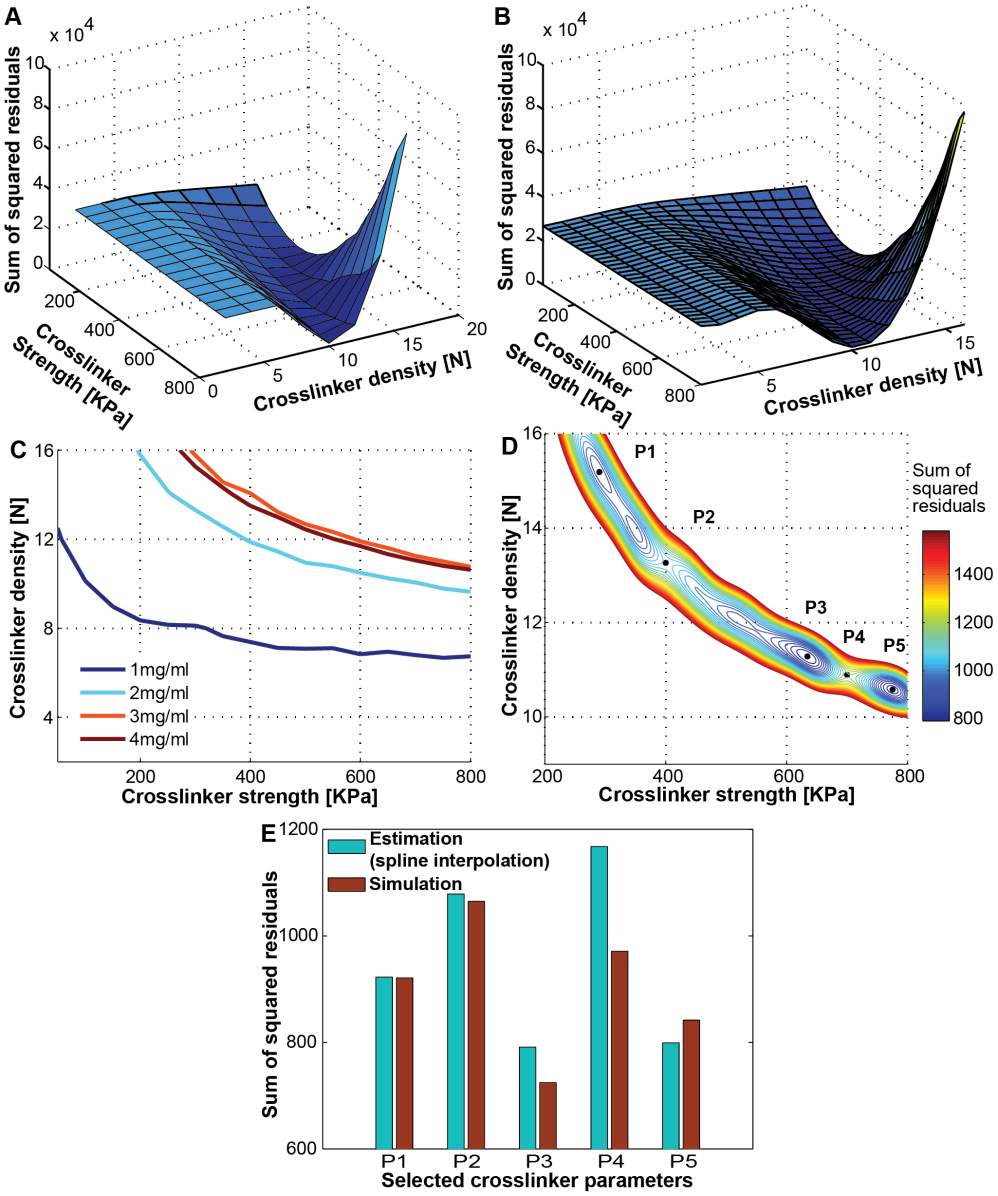
Finding the best-fit crosslinker parameter values. (A) The sum of squared residuals (SSR) between the shear elastic moduli data from Stein et al. [30] and our simulations (figure 3D), for four different collagen densities. (B) The spline interpolation of (A) using the half of the prior parameter intervals provides smoother surface of the sum of squared residuals. (C) Intersection lines of experimental data plane with simulation surfaces of figure 3D. (D) Zoomed-in contour plot of the 7^th^ spline interpolation of the SSR around the minimum value to the two times of the minimum value. Five points were selected for comparison: P1 (290.23 KPa for crosslinker strength, 15.19N for crosslinker density), P2 (400 KPa, 13.27N), P3 (634.38 KPa, 11.28N), P4 (700 KPa, 10.89N), and P5 (775.39 KPa, 10.58N). (E) Validation for the best-fit crosslinker parameter values. We have compared spline interpolated SSR estimation values with calculated SSR value using simulation results for these 5 selected crosslinker parameter points. 30 independent simulations were run to calculate SSR values. The P3 crosslinker parameter values were chosen as the best-fit value because both spline estimated SSR and calculated SSR using simulation are the lowest value.

Assuming that the crosslinker characteristics remain the same when we change the collagen density alone, we can fit for their values using experimental data. We searched for the optimal crosslinker parameter values by minimizing the difference between simulation data and experimental data. The elastic modulus of shear experiments is 3.03 Pa for 1 mg/ml, 44.50 Pa for 2 mg/ml, 97.38 Pa for 3 mg/ml, and 123.5 Pa for 4 mg/ml from experimental data [30]. Figure 4A shows the sum of squared residuals (SSR) between the simulated and experimental shear moduli using 4 collagen densities, as a surface in the two independent variable space of crosslinker density and crosslinker strength. The crosslinker density and strength parameter at the minimum SSR value corresponds to the optimal parameter value. We performed iterative spline interpolation by halving the crosslinker parameter intervals. Figure 4B shows an intermediate, smoother interpolated SSR surface plot. Figure 4C shows the intersection lines between simulated shear modulus surfaces and experimental elastic modulus data of 4 collagen densities. These lines correspond to the constraints on the crosslinker parameters that could produce the experimental shear moduli. The iterative spline interpolation built an estimated surface plot of the SSR after the 7^th^ interpolation. Figure 4D shows the zoomed-in contour plot of SSR near the minimum, from which we compared 5 points. P1 is one SSR minimum, where crosslinker strength is less than 400 KPa, P5 is another SSR minimum, where crosslinker strength is larger than 700 KPa, and P3 is the lowest SSR point over all queried crosslinker parameter space. P2 and P4 are transition points in between these local minima, where crosslinker strength is 400 KPa and 700 KPa, respectively. Figure 4E showed the SSR value from both estimated values by spline interpolation and calculated value by simulations of the five selected crosslinker parameter values. P3 corresponds to the lowest SSR for both estimated and calculated values, and thus we used this crosslinker strength (634.38 KPa) and crosslinker density (11.18N) combination as the best-fit crosslinker parameters. As both increasing the crosslinker strength and density increases the network modulus, it is expected that the crosslinker strength and density have a reciprocal relationship, which is reflected in the shape of the contour plot in Figure 4D.

### Shear and tensile tests in small strain regions for various collagen densities validate the model

**Figure 5:**
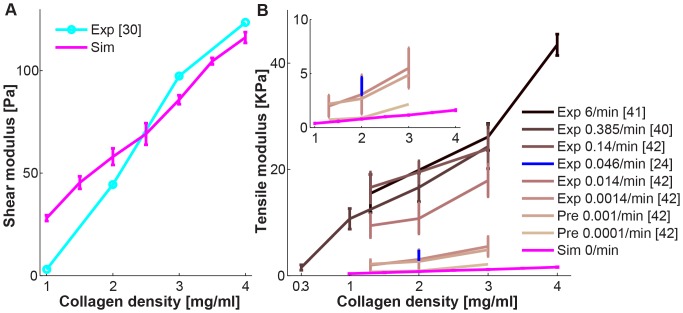
Validation of the best-fit crosslinker parameter values. (A) Shear modulus of simulation results (Sim) using the best-fit crosslinker parameter values and elastic modulus (G′) in shear experiments (Exp) from Stein et al. [30]. 5 independent runs were simulated for seven different collagen densities (1, 1.5, …, 4 mg/ml using a 0.5 mg/ml increment). (B) Tensile modulus of various strain rate experiments, experiments from Provenzano et al. [41], Roeder et al. [40], Riching et al. [24], Lopez-Garcia et al. [42], predicted values (Pre) using a power-law fitting from Lopez-Garcia et al. [42], and simulation results using the best-fit crosslinker parameter values. Inset figure is magnified view of our experimental data of 2 mg/ml collagen gels at very slow train rate of 0.046/min.

Using these best-fit crosslinker parameter values, we simulated shear and tensile stretching tests for seven different collagen densities (1–4 mg/ml, 0.5 mg/ml increment) in a small strain region (0–0.1 strain, 0.01 strain step size). Figure 5A compares simulated shear moduli with the experimental elastic moduli (G′) of shear tests [30], showing a good agreement in the middle collagen densities (2 and 3 mg/ml). The difference between simulations and experiments increases at high collagen density and low collagen density. In figure 5B, we compiled all *in vitro* tensile moduli for various collagen densities with different strain rates that we could find in the literature [40]–[42] and from our experiments [24]. In addition, we also added the predicted tensile modulus using a power-law fitting [42]. Not surprisingly, from the experimental data, we see a significantly decrease in the tensile moduli at lower strain rates, where the viscous effects are weak. Recall that we built our fiber network model under the ideal elastic assumption and that we determined the crosslinker parameters with elastic modulus of shear data. It therefore comes as no surprise that the simulated tensile moduli are lower than experimental tensile measurements, but in good agreement with the predicted value of a very low strain rate of 0.0001/min [42]. These results suggest that our model is functioning in a realistic manner for small strain rate regimes, which should resemble cell-fiber strain *in vivo* conditions.

### Shear and tensile tests in larger strain regimes suggest the key role of network geometries

**Figure 6:**
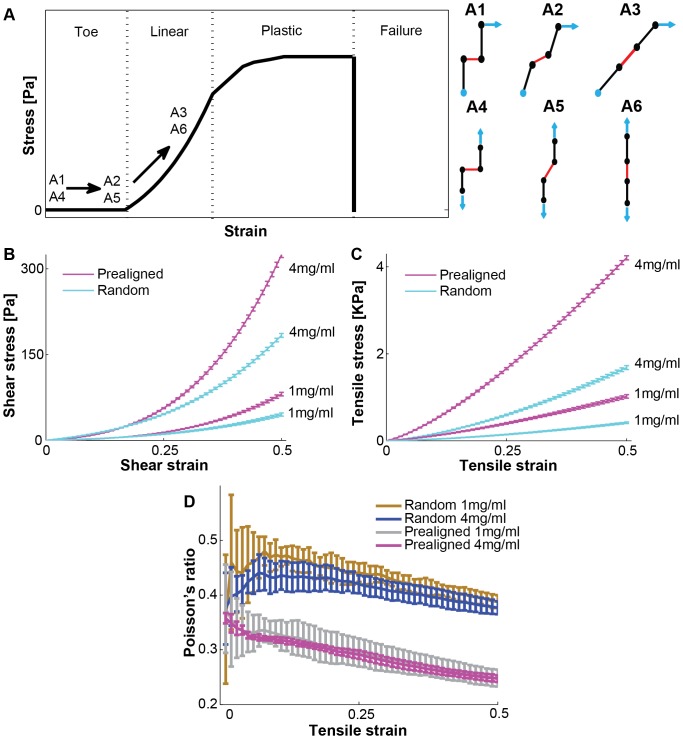
Stress-strain curves from small strain toe region to medium strain linear region. (A) Schematic stress-strain curve to illustrate toe, linear, plastic, and failure region. A collagen fiber network is soft at the small deformation state, but stiff at the large deformation state because realigning fibers through crosslinkers play a pivotal role in the strain stiffening. Realignment illustration of fiber network model for shear test (A1: zero strain, A2: small strain, A3: medium strain) and tensile test (A4: zero strain, A5: small strain, A6: medium strain). Black solid lines represent collagen fibers and red solid lines are crosslinkers. Black dots represent beads which can have elastic connection with other beads. Light blue solid arrows represent force vectors, and a light blue dot represents anchored fixed beads for shear test. Simulated stress-strain curves of shear test (B) and tensile test (C) for two different collagen densities: 1, 4 mg/ml and two different network geometries: prealigned network and random network using the best-fit crosslinker parameter values. The errorbars are standard deviation from the mean in 5 independent simulations. We simulated up to 0.5 strain with a 0.01 strain step size. (D) Poisson's ratio of tensile tests in (C) for both random and prealigned networks.

The typical textbook illustration of a complete tensile stress-strain curve for a collagen network consists of a small strain toe region with little stress change, a medium strain linear region, a large strain plastic region, and finally the failure region when the network breaks (figure 6A). To clearly illustrate differences between shear and tensile experiments in the transition between the small and large strain response regions, we used a simple schematic fiber network model, two collagen fibers with one crosslinker (Figure 6: A1–A6). Figures A1–A3 illustrate a shear test, where the light blue bead at the bottom is anchored and the top bead is moved to the right. From the initial relaxed state at zero strain (A1), the fibers rotate and displace at low strain (A2), then the fiber network becomes aligned in the large shear strain (A3). Figures A4–A6 illustrate a tensile test, from the initial relaxed state at zero strain (A4), the fibers also rotate and displace at low strain (A5), to completely align at larger strain (A6). Figure 6A illustrates our understanding of how the transition from toe to linear region occurs in the elastic fiber network model: the small strain toe region is where the applied force rotates and aligns the fibers in the network (from A1 to A2 in the shear test and from A4 to A5 in the tensile test); the medium strain linear region is when the fiber network is completely aligned; fiber and crosslinker stretching then is responsible for the network response (from A2 to A3 in the shear test and from A5 to A6 in the tensile test); the large strain plastic region is where individual fibers are damaged irreversibly, and the last failure region is where either the collagen fibers or the crosslinkers are broken. In the shear test, the fibers do not align with the direction of the external force, but in the tensile test the fibers align with the direction of the force. This difference is the reason for a longer toe region in the shear test because the fibers, even when they are aligned, can still rotate under external force.

Using the best-fit crosslinker parameter values, we examined the fiber network in larger strain regions, and simulated both shear and tensile tests by applying strain from 0.01 to 0.5, with 0.01 strain step increment. We compared two different network geometries (random *vs*. prealigned) for two different collagen densities (1 and 4 mg/ml). Figure 6B and 6C show the stress-strain curves for shear and tensile tests (corresponding movies S1–S6). In shear tests, the stress-strain curves of the prealigned network and the random network are quite similar at the small strain toe region, while at the larger strain region the prealigned network is much stiffer than the random network. In tensile tests, the prealigned fiber networks are stiffer than the random fiber network in both the small strain and larger strain regions. The fiber network geometry and applied force direction are the key factor to alter the transition from toe to linear region.

We also calculated Poisson's ratio for tensile tests in figure 6C by the ratio of lateral strain to longitudinal strain. To calculate the lateral strain, we sampled 9 different z-axis points (50, 75, 100, 125, 150, 175, 200, 225, 250 µm in height), and fitted a rectangular lateral strain box by averaging fibers located at the simulation box boundary area, which is 2.5% of the total number of fibers. Figure 6D presented Poisson's ratio of four different tensile tests in figure 6C from 0.01 strain to 0.5 strain. The ratio started around 0.36 at the small strain (0.01) for all four different test cases, and then reaches 0.38 for random fiber network and 0.25 for prealigned fiber network at the large strain (0.5). The network geometrical structure strongly alters Poisson's ratio, while collagen density weakly alters Poisson's ratio. To clearly address each parameter effect on Poisson's ratio further, we simulated 7 different crosslinker strength values (200, 300, 400, 500, 600, 700, 800 KPa), 8 different crosslinker density values (2, 4, 6, 8, 10, 12, 14, 16N), 2 different collagen densities (1, 2 mg/ml), and 2 different fiber network structures (random, prealigned) for tensile test, which is total 224 different test conditions. In each condition, we run 5 independent simulation runs from 0.01 to 0.5 strain. Figure S2 shows contour plot of Poisson's ratio for four different parameter effects, collagen density, fiber network structure, crosslinker density, and crosslinker strength. Network geometrical structure and crosslinker density strongly alter Poisson's ratio, while collagen density and crosslinker strength weakly influence Poisson's ratio.

### Local deformation simulation shows quantitative rapid stress and deformation propagation in the fiber network

**Figure 7:**
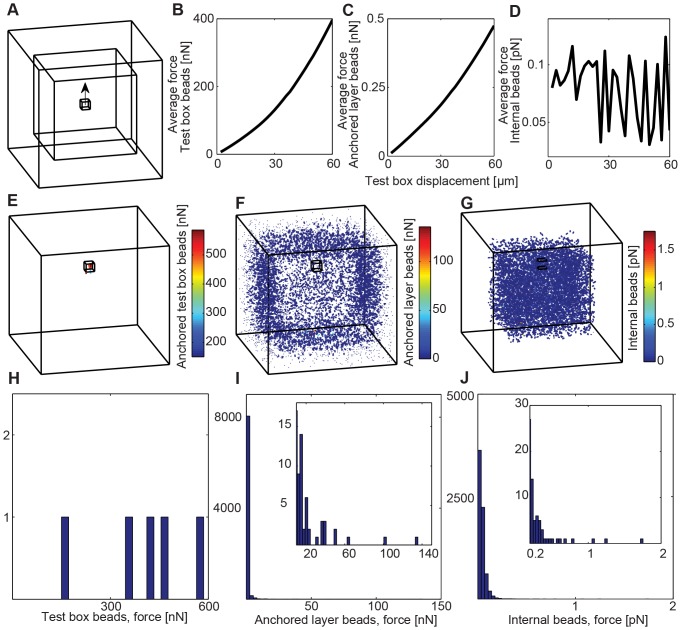
Simulation of a local deformation test using the calibrated collagen model of 2 mg/ml. (A) A cubic test box (20 µm×20 µm×20 µm) is located at the center of the simulation box (300 µm×300 µm×300 µm). All beads in the test box are anchored and displaced by 60 µm in the z-direction (black arrow) with a 2 µm displacement step size for 30 steps. Beads in the outer layer of the simulation box (within 50 µm of all the box sides) are anchored. All fiber-beads are initially at equilibrium before the test box is displaced. Average force value was calculated at the quasi-equilibrium state after each displacement step. Average force value of all beads in the test box (B), anchored layer (C), and internal box (D) over 30 displacement steps. Force vectors at the quasi-equilibrium state of 60 µm displacement in the test box (E), anchored layer (F), and internal box (G). Each colorbar shows force scale in the figure. Force histogram at the quasi-equilibrium state of 60 µm displacement in the test box (H), anchored layer (I), and internal box (J). Inset images of figure I and J are magnified views to illustrate the tails of distribution at larger force values.

To study the effect of a local force such as might be exerted by a migrating cell in the collagen network, we performed a local deformation test. From an initial state, we anchored and fixed all beads in the outer-layer of the simulation box within 50 µm from the top, bottom and sides of the 300×300×300 µm^3^ simulation box. We picked a 20×20×20 µm^3^ test cube, located at the center of the simulation box, and displaced the cube 60 µm in the z-direction in 2 µm steps (figure 7A). Figures 7B–D are the averaged force measurements for the text box, the anchored layer beads, and the interior beads. Note the averaged force for the anchored beads is in the order of nN, while that for interior beads is in order of pN. To demonstrate how a local force is propagated to the whole network, we plotted force vectors in time, by assuming adiabatic displacement of the test box in the z-direction (movie S7). Figure 7E–G show the force vectors after energy minimization, illustrating the distribution of the local deformation through the network. Figures 7H–J show the histogram of force values of all beads in the test box, anchored beads, and the interior beads of the simulation box. We see that stress generated by local deformation starts around the test box and quickly spreads through the neighboring fibers and across the fiber network. This result agrees with the 3D traction measurements by Legant et al. [45], where traction forces of NIH 3T3 fibroblast in a 3D elastic hydrogel matrix were in the range of 100–5000 Pa. On the leading surface of our test box in the local deformation simulation, this force is equal to 40–2000 nN, whereas the force in our simulation was in the order of 100 nN. This agreement supports that our collagen model mechanics is in the range of a migrating cell.

## Discussion

We have developed an elastic fiber network model (beads-and-spring) of aligned and random collagen networks that contains explicit elastic inter-fiber crosslinkers. The phenomenological crosslinker model allows us to adjust the fiber network connectivity and strength, so that we can quantitatively examine the effect of diverse crosslinker parameters on the mechanical properties of fiber network system. We used experimental single fiber parameters and elastic modulus data in shear experiments to find the best-fit crosslinker parameter values by assuming that the viscous effect of collagen fiber network is negligible at the time scale of cell migration. Using these parameters, we performed further shear and tensile simulations to validate the model, and demonstrate the model potential in responding to local deformations. Overall our 3D mechanical elastic fiber collagen model is a useful tool to identify network outcomes of different matrix properties and for future interface with cell and tumor 3D models.

One interesting result of our simulations is the clear demonstration that network property depends more sensitively on the network structure than other parameters, such as collagen density. Thus, the initial fiber orientation (prealigned *vs* random) strongly influences the mechanical property of the fiber network, directly related to the strain direction and fiber realignment. Our elastic fiber network model can capture strain stiffening, including the transition from toe to linear response regions. This observation is in good agreement with the experimental stress-strain curves [40], [46]. It also recapitulates the strain-stiffening characteristic of non-affine fiber networks [28], [30].

Real collagen gels would eventually break in rheometry tests, at around 0.6 strain in nonsinusoidal stress-strain tests of 2 mg/ml collagen [40], and at around 0.2 strain in sinusoidal stress-strain tests of 0.9 mg/ml collagen [46]. To capture this mechanical property, we should allow the crosslinkers or the fibers in our model to break. Buehler et al. [47] showed the mechanical properties and breakage points of intra-fiber (or inter-fibril) crosslinkers in collagen type I, and how the breakage strain point varies by inter-fibril crosslinker densities, up to 0.45 strain. We could extend our model to incorporate this feature of collagen fibers. Presently the goal for our model is as a building block for integration with mechanical cell models (e.g. [48]). The amount of strain by migrating cells in a tissue is relatively small, not in the failure region of the stress-strain curve but rather in the small strain region [24]. Our model is compatible with the spatiotemporal scale of collagen remodeling resulting from a migrating cell.

Our model shows that local stress propagates and decays through the fiber network. In Münster et al. [46], a collagen fiber network reaches the quasi-equilibrium state almost instantaneously in the rheology measurement. Therefore, we can use the quasi-equilibrium state of the elastic fiber network model as an instantaneous mechanical response of a local deformation by a protruding or migrating cell. Interestingly we see that the distribution of the stress is not homogeneous, resembling the stress distribution on other inhomogeneous media, e.g., stress in granular media or in earth rocks. Furthermore, even when the fiber network has reached the quasi-equilibrium state, there is still residual stress, albeit small, near the initial deformation. We have further demonstrated that repeated local deformation results in accumulation of stress in the fiber network. These results suggest that more deformation, such as might occur with collective cellular migration or growth of multicellular tumors, a significant amount of stress would accumulate in the fiber network, leading to a large scale alignment of the fiber network.

Lastly, although much of the ECM in the breast is collagen type I, a real ECM is a complex mixture of different ECM protein fibers. Even a collagen matrix can be a mixture of different collagen types, including type I, type IV, type V and others. For example, it has been shown that network stiffness significantly decreases in matrices containing more collagen type V [49]. This difference could be due to altered non-covalent interactions in collagen mixtures. Our modeling method would still work well by fitting for the equivalent crosslinker parameters. Also collagen fiber networks of *in vitro* or *in vivo* condition are heterogeneous, and the typical diameter of a fiber increases as the collagen density increases [49]. Many other physical and chemical factors also contribute to the mechanical properties of collagen fiber network, such as gel thickness [50] and pH [40]. Our elastic fiber network model is a simple and generic model that allows for expansion and inclusion of more complicated parameters and conditions to simulate more realistic ECM environment, including heterogeneous fiber length and thickness. Even the most carefully controlled protocol for generating *in vitro* collagen would generate a gel with a distribution of collagen fiber width and length. As fiber width would change the fiber modulus, our model network of fibers with identical length and width might be a factor contributing to the discrepancy between our calibrate model and experimental data. Despite this, the model serves as an efficient and accurate starting point to simulate how fiber network and connectivity parameters interact with cell rheology parameters, how locally deformed fibers alter the global fiber network structure, and how the realigned and deformed fiber networks influence on invasive cellular behaviors.

## Methods

### Collagen gel preparation and second harmonic imaging

Collagen gels were prepared as previously described [51] and cast in a dogbone-shaped mold with dimensions described in Roeder et al. [40]. Gels were allowed to polymerize at 37°C overnight. To generate aligned collagen, gels were removed from the mold, and mechanically strained to 30% using a custom fabricated device. This device was also designed to fit the stage of a multiphoton microscope to facilitate second harmonic generation (SHG) imaging of collagen following the application of strain. Images of collagen gels were acquired with WiscScan software and a Nikon 40× Apo water immersion lens (Numerical Aperture, N.A. 1.15 and Working Distance, W.D. 0.61).

### Collagen fiber network simulations

The three dimensional off-lattice collagen fiber network model was implemented by C++ programing language and compiled by gnu C++ compiler. All simulations were run on Euler cluster at the Wisconsin Applied Computing Center. Analyses of simulation data and making of simulation movies were done using MATLAB 2013b. Prototype code was implemented by both Matlab and C++ language and was tested on Octan and Carina clusters at Georgia State University.

## Supporting Information

Figure S1 **Anchored depth effect on shear simulation tests of 1 mg/ml collagen density, 100 KPa crosslinker strength, eight different crosslinker densities (2, …, 16N), and five different anchored depths (10, 20, 50, 100, 200 µm), corresponding to the simulation box size 200 µm (length) ×200 µm (width) ×220, 240, 300, 400, 600 µm (height).** Shear modulus was calculated from the stress-strain curve in small strain region (0–0.1strain, 0.01 strain step increment). Three independent runs were simulated and then averaged. The half of collagen fiber length (50 µm) is the minimum enough anchored depth, and any larger depths did not significantly different from 50 µm depth case. However, the smaller anchored depth than 50 µm showed reduced shear modulus, meaning less number of fibers anchored for the given collagen density.
(TIF)

Figure S2 **Contour plot of Poisson's ratio for tensile tests at 0.5 strain.** Random fiber network for (A) 1 mg/ml, (B) 2 mg/ml, Prealigned fiber network for (C) 1 mg/ml, (D) 2 mg/ml. Simulation box is 200 µm (length) ×200 µm (width) ×300 µm (height) with the anchored top 50 µm and bottom 50 µm. We simulated 7 different crosslinker strength values (200, 300, 400, 500, 600, 700, 800 KPa), 8 different crosslinker density values (2, 4, 6, 8, 10, 12, 14, 16N), two different collagen densities (1, 2 mg/ml), and two different fiber network structures (random and prealigned) for tensile test, which correspond to 224 different test conditions. In each condition, we run 5 independent simulation runs from 0.01 to 0.5 strain with 0.01 strain step size.
(TIF)

Movie S1 **Shear test simulation movie for two different collagen fiber network geometries: random fiber network** ***vs.*** **prealigned fiber network.** The collagen density for this test simulation is 1 mg/ml and deformed the simulation box from 0 strain to 0.5 strain, using a 0.01 strain step increment. Simulation box size is 200 µm (length) ×200 µm (width) ×300 µm (height). The top 50 µm and bottom 50 µm of the box is anchored area. All fiber-beads in the anchored area are fixed and then the top anchored area is deformed to y-direction. Each snapshot image in the movie is taken at the quasi-equilibrium state after each 0.01 strain step (2 µm) was applied.
(MP4)

Movie S2 **Force distribution movie for the 1 mg/ml shear simulation in movie S1.** In each quasi-equilibrium state, forces of anchored fiber-beads and forces of internal deformable fiber-beads were presented by vectors. Note the stress for anchored beads is in the order of nN, while the stress for the internal beads is in the order of pN. We plot the force vectors of anchored beads twice as thick as those of internal beads. The histograms plot force distribution in anchored and internal beads.
(MP4)

Movie S3 **Shear test simulation movie for two different collagen densities: 1 mg/ml** ***vs.*** **4 mg/ml.** The collagen fiber geometry for this test simulation is random fiber network and deformed the simulation box from 0 strain to 0.5 strain, using a 0.01 strain step increment. Simulation box size is 200 µm (length) ×200 µm (width) ×300 µm (height). The top 50 µm and bottom 50 µm of the box is anchored area. All fiber-beads in the anchored area are fixed and then the top anchored area is deformed to y-direction. Each snapshot image in the movie is taken at the quasi-equilibrium state after each 0.01 strain step (2 µm) was applied.
(MP4)

Movie S4 **Tensile test simulation movie for two different collagen fiber network geometries: random fiber network** ***vs.*** **prealigned fiber network.** The collagen density for this test simulation is 1 mg/ml and deformed the simulation box from 0 strain to 0.5 strain, using a 0.01 strain step increment. Simulation box size is 200 µm (length) ×200 µm (width) ×300 µm (height). The top 50 µm and bottom 50 µm of the box is anchored area. All fiber-beads in the anchored area are fixed. Each snapshot image in the movie is taken at the quasi-equilibrium state after each 0.01 strain step (1 µm to z at the top and 1 µm to -z at the bottom) was applied.
(MP4)

Movie S5 **Force distribution movie for the 1 mg/ml tensile simulation in movie S4.** In each quasi-equilibrium state, forces of anchored fiber-beads and forces of internal deformable fiber-beads were presented by force vectors. Note the stress for anchored beads is in the order of nN, while the stress for the internal beads is in the order of pN. We plot the force vectors of anchored beads twice as thick as those of internal beads. The histograms plot force distribution in anchored and internal beads.
(MP4)

Movie S6 **Tensile test simulation movie for two different collagen densities: 1 mg/ml** ***vs.*** **4 mg/ml.** The collagen fiber geometry for this test simulation is random fiber network and deformed the simulation box from 0 strain to 0.5 strain, using a 0.01 strain step increment. Simulation box size is 200 µm (length) ×200 µm (width) ×300 µm (height). The top 50 µm and bottom 50 µm of the box is anchored area. All fiber-beads in the anchored area are fixed, and then the top anchored area is deformed to z-direction and the bottom anchored area is deformed to –z-direction. Each snapshot image in the movie is taken at the quasi-equilibrium state after each 0.01 strain step (1 µm to z at the top and 1 µm to -z at the bottom) was applied.
(MP4)

Movie S7 **Local deformation simulation movie for a random fiber network of 2 mg/ml (** **Figure 7** **).** The test local deformed box (20 µm×20 µm×20 µm) is located at the center of the simulation box (300 µm×300 µm×300 µm). All beads in the test box are anchored and displaced in the z-direction with a 2 µm displacement step size for 30 steps. All beads are anchored and fixed in the outer layer of the simulation box (within 50 µm of all the box sides). Force vectors in test box, anchored layer, and internal box at quasi-equilibrium after each 2 µm displacement are separately presented in the top row. Note that the color bars indicate that the forces on anchored beads are in the order of nN, and those for the interior beads are in the order of pN. The bottom row shows the histograms of the forces in the test box, the anchored layer, and the internal box. Insets show magnified view of the tails of distribution at larger force values.
(MP4)
