# Supplementary figures and images for: A Three-Dimensional Computational Model of Collagen Network Mechanics
Source: PLoS One. 2014 Nov 11;9(11):e111896. doi: 10.1371/journal.pone.0111896 (PMC4227658; doi:10.1371/journal.pone.0111896)

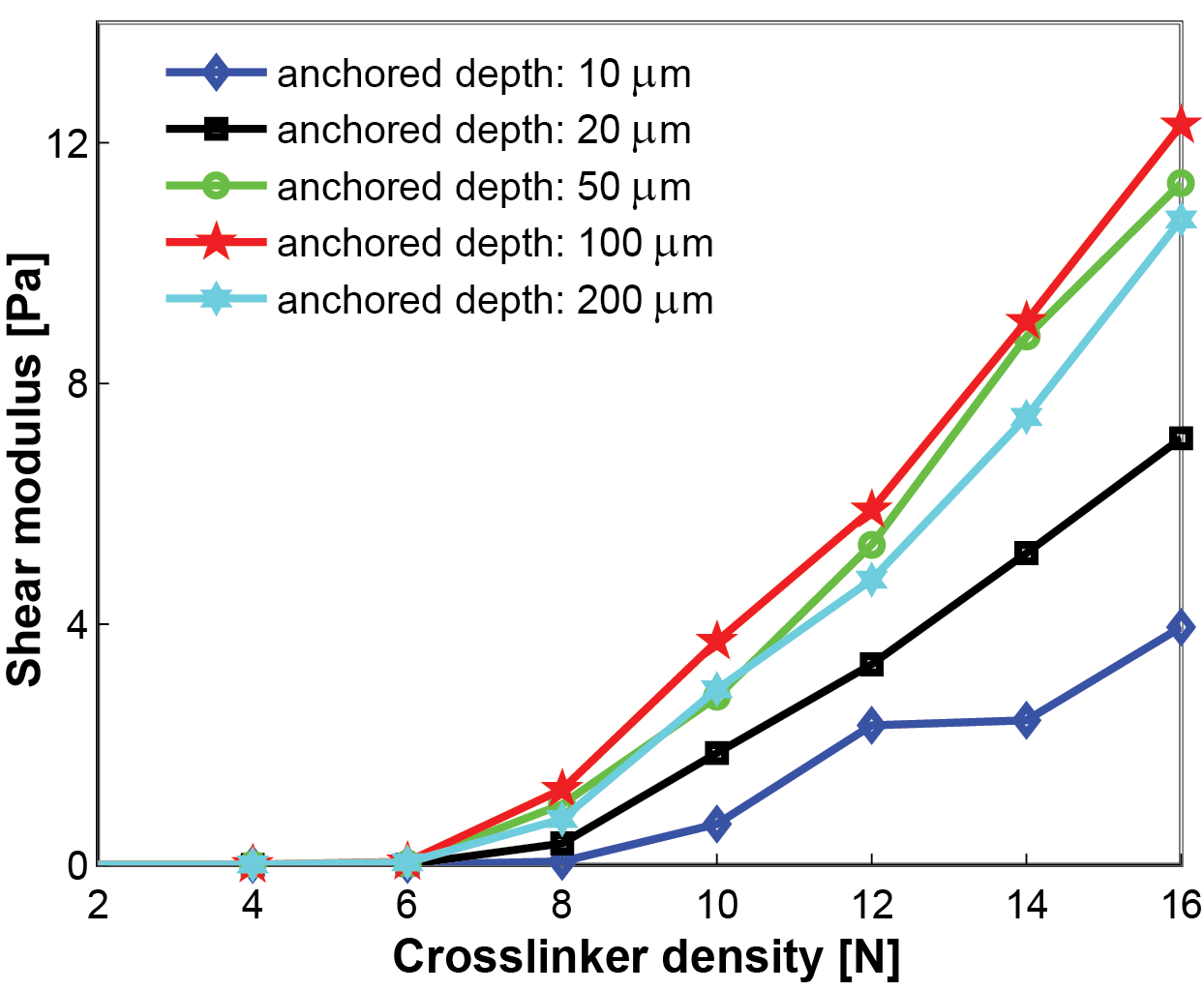

Supplement: Figure S1 — Anchored depth effect on shear simulation tests of 1 mg/ml collagen density, 100 KPa crosslinker strength, eight different crosslinker densities (2, …, 16N), and five different anchored depths (10, 20, 50, 100, 200 µm), corresponding to the simulation box size 200 µm (length) ×200 µm (width) ×220, 240, 300, 400, 600 µm (height). Shear modulus was calculated from the stress-strain curve in small strain region (0–0.1strain, 0.01 strain step increment). Three independent runs were simulated and then averaged. The half of collagen fiber length (50 µm) is the minimum enough anchored depth, and any larger depths did not significantly different from 50 µm depth case. However, the smaller anchored depth than 50 µm showed reduced shear modulus, meaning less number of fibers anchored for the given collagen density. (TIF) [file pone.0111896.s001.tif]

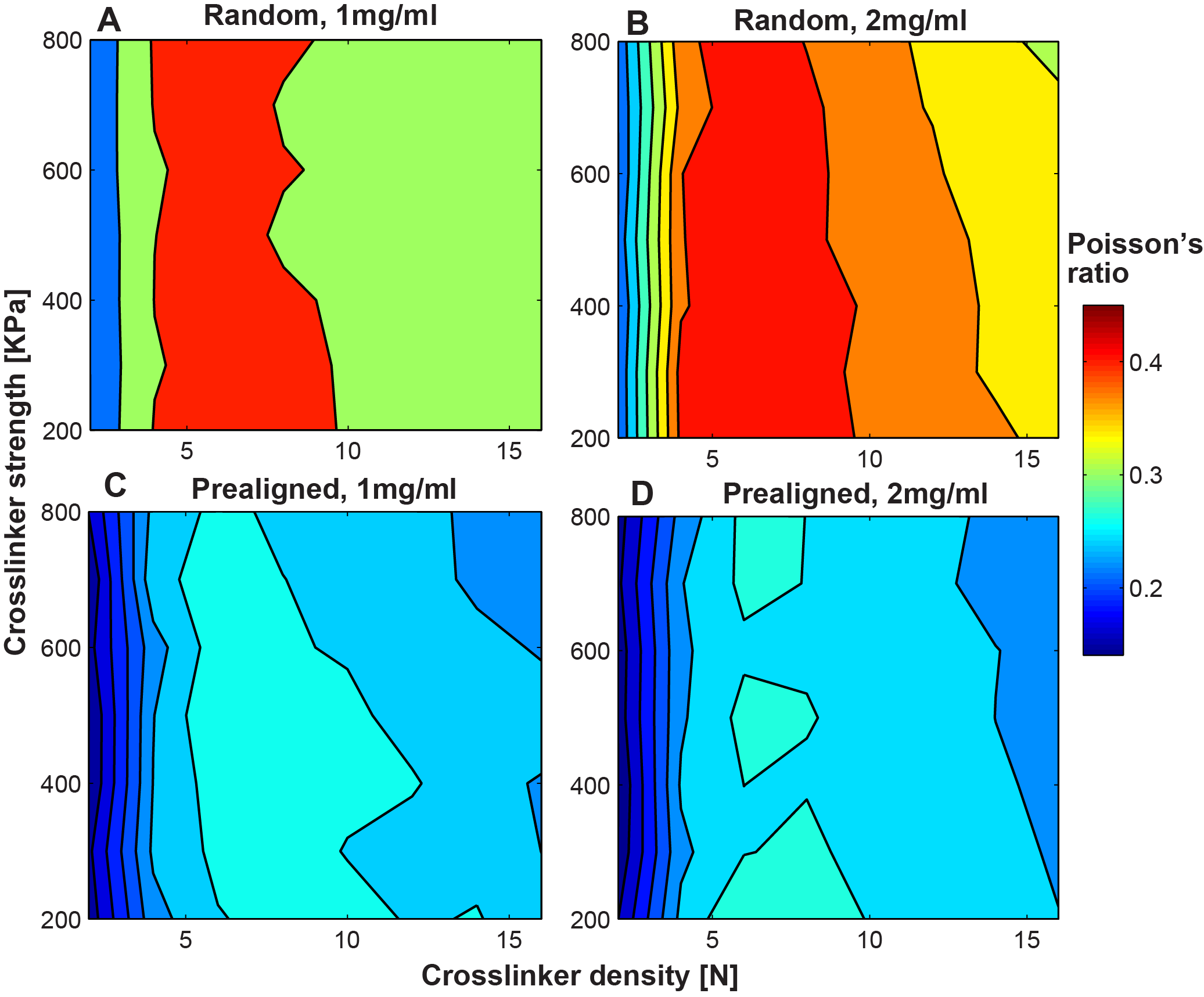

Supplement: Figure S2 — Contour plot of Poisson's ratio for tensile tests at 0.5 strain. Random fiber network for (A) 1 mg/ml, (B) 2 mg/ml, Prealigned fiber network for (C) 1 mg/ml, (D) 2 mg/ml. Simulation box is 200 µm (length) ×200 µm (width) ×300 µm (height) with the anchored top 50 µm and bottom 50 µm. We simulated 7 different crosslinker strength values (200, 300, 400, 500, 600, 700, 800 KPa), 8 different crosslinker density values (2, 4, 6, 8, 10, 12, 14, 16N), two different collagen densities (1, 2 mg/ml), and two different fiber network structures (random and prealigned) for tensile test, which correspond to 224 different test conditions. In each condition, we run 5 independent simulation runs from 0.01 to 0.5 strain with 0.01 strain step size. (TIF) [file pone.0111896.s002.tif]
